# Adopting the RE-AIM analytic framework for rural program evaluation: experiences from the Advance Care Planning via Group Visits (ACP-GV) national evaluation

**DOI:** 10.3389/frhs.2024.1210166

**Published:** 2024-03-25

**Authors:** Monica M. Matthieu, Laura D. Taylor, David A. Adkins, J. Silas Williams, Bo Hu, Ciara M. Oliver, Jane Ann McCullough, Mary J. Mallory, Ian D. Smith, Jacob T. Painter, Songthip T. Ounpraseuth, Kimberly K. Garner

**Affiliations:** ^1^ U.S. Department of Veterans Affairs Medical Center, Central Arkansas Veterans Healthcare System, HSR&D Center of Innovation: Center for Mental Healthcare & Outcomes Research, Little Rock, AR, United States; ^2^ School of Social Work, Saint Louis University, Saint Louis, MO, United States; ^3^ U.S. Department of Veterans Affairs Medical Center, Central Arkansas Veterans Healthcare System, Geriatric Research, Education and Clinical Center, Little Rock, AR, United States; ^4^ Division of Pharmaceutical Evaluation & Policy, College of Pharmacy, University of Arkansas for Medical Sciences, Little Rock, AR, United States; ^5^ Department of Biostatistics, Fay W. Boozman College of Public Health, University of Arkansas for Medical Sciences, Little Rock, AR, United States; ^6^ Department of Psychiatry, College of Medicine, University of Arkansas for Medical Sciences, Little Rock, AR, United States

**Keywords:** veteran, advance care planning, advance directive, implementation science, RE-AIM framework, United States Department of Veterans Affairs

## Abstract

**Introduction:**

To support rigorous evaluation across a national portfolio of grants, the United States Department of Veterans Affairs (VA) Office of Rural Health (ORH) adopted an analytic framework to guide their grantees' evaluation of initiatives that reach rural veterans and to standardize the reporting of outcomes and impacts. Advance Care Planning via Group Visits (ACP-GV), one of ORH's Enterprise-Wide Initiatives, also followed the Reach, Effectiveness, Adoption, Implementation, and Maintenance (RE-AIM) framework. ACP-GV is a national patient-centered intervention delivered in a large, veterans integrated healthcare system. This manuscript describes how RE-AIM was used to evaluate this national program and lessons learned from ORH's annual reporting feedback to ACP-GV on their use of the framework to describe evaluation impacts.

**Methods:**

We used patient, provider, and site-level administrative health care data from the VA Corporate Data Warehouse and national program management databases for federal fiscal years (FY) spanning October 1, 2018–September 30, 2023. Measures included cumulative and past FY metrics developed to assess program impacts.

**Results:**

RE-AIM constructs included the following cumulative and annual program evaluation results. ACP-GV reached 54,167 unique veterans, including 19,032 unique rural veterans between FY 2018 to FY 2023. During FY 2023, implementation adherence to the ACP-GV model was noted in 91.7% of program completers, with 55% of these completers reporting a knowledge increase and 14% reporting a substantial knowledge increase (effectiveness). As of FY 2023, 66 ACP-GV sites were active, and 1,556 VA staff were trained in the intervention (adoption). Of the 66 active sites in FY 2023, 27 were sites previously funded by ORH and continued to offer ACP-GV after the conclusion of three years of seed funding (maintenance).

**Discussion:**

Lessons learned developing RE-AIM metrics collaboratively with program developers, implementers, and evaluators allowed for a balance of clinical and scientific input in decision-making, while the ORH annual reporting feedback provided specificity and emphasis for including both cumulative, annual, and rural specific metrics. ACP-GV's use of RE-AIM metrics is a key step towards improving rural veteran health outcomes and describing real world program impacts.

## Introduction

In supporting rigorous evaluation across a national portfolio of grants, the United States Department of Veterans Affairs (VA) Office of Rural Health (ORH), an intramural grant funding office, adopted an evidence based analytic framework. ORH did this to improve their grantees' evaluation of innovative initiatives that reach rural veterans and to standardize the reporting of outcomes and impacts. One ORH funded Enterprise-Wide Initiative, the National Advance Care Planning via Group Visits (ACP-GV) Program, was an early adopter of the Reach, Effectiveness, Adoption, Implementation, and Maintenance (RE-AIM) analytic framework. RE-AIM, a widely used framework (1), outlines domains of importance to be considered in developing metrics that guide evaluation and provides summarized information relevant to demonstrating impact of the program under study. In the following section, we define advance care planning, describe the use of a group modality used to deliver advance care planning, and the requisite training and tools to support program development and implementation of ACP-GV in VA healthcare settings.

### What is advance care planning via group visits?

Advance care planning is a process of identifying personal values and preferences and designating individuals who can support health care decision making when unable to communicate these wishes for themselves. Advance care planning is associated with increased likelihood that health care will align with patient values, decrease caregiver stress, and reduce health care spending at the end of life (2, 3). At times, these wishes are documented in an advance directive, which is a legal document describing an individual's advance care planning values and preferences (4). Having an up-to-date written advance directive (4) provides guidance for veterans to support their future communication on this topic with families, caregivers, trusted others, and their care team(s).

The need for preparatory health care planning for all veterans, regardless of health status, is great. At the policy level, advance care planning is a mandated process within VA healthcare settings and is required by The Joint Commission (5) and the Patient Self Determination Act (6). At the VA organizational level, a new revised organizational policy provides detailed guidance for health care executives and clinicians to meet these requirements (7). However, based on community advance directive completion rates of approximately 26% (8) we estimate that 6.7 of the 9.07 million veterans currently enrolled in the VA healthcare system (9) may not have an advance directive. These veterans are at-risk of receiving care not aligned with their preferences or being hospitalized with their preferences unknown by others.

As one way to address this gap, ACP-GV promotes having a conversation facilitated by a clinician (e.g., physician, social worker, etc.) regarding a veterans' future care preferences. ACP-GV is unique in that this discussion is conducted in a group setting which may be comprised of exclusively veterans, caregivers of veterans, and/or those they trust. This 60-minute patient-centered intervention assembles small groups of up to ten participants, in person or virtually, with a group facilitator. The facilitator guides a group discussion, including sharing of life experiences and values. This sharing among veterans is a hallmark of the group; the camaraderie known to be developed during military service increases the impact and peer-to-peer influence of the group. At the end of the group discussion, ACP-GV encourages all participants to identify a “next step” to take in the process of planning for their future care needs. The next step, or goal, allows the facilitator to follow-up with the participant to answer any additional questions or to help complete necessary documentation.

In order to prepare clinical staff to lead this structured conversation, the National ACP-GV Program, offers prospective facilitators training in the group curriculum called “Facilitator Training.” The content includes an orientation, foundational review of the ACP-GV intervention and its essential elements, benefits of using the group modality, key concepts in advance care planning, and Motivational Interviewing concepts that support goal setting (10). After completing Facilitator Training, trained staff may use the curriculum in any setting where groups are common or at a newly established practice in their respective setting.

For some clinicians, program development, implementation, and evaluation may require additional training to support the more administrative functions such as ongoing marketing, veteran, provider, and/or clinic recruitment, referral and workflow management, staff development, and leadership of the program. This is the role of the ACP-GV site lead(s). “Site Lead Training” is offered to help guide staff to embed the program within a clinic (e.g., primary care) to a professional group of clinicians (e.g., social workers) across an entire department, practice setting (e.g., inpatient, outpatient or residential), medical center, or across a network of primary care, urgent care, or community-based clinics.

In this training, the core functions for site leads are reviewed. They are: (1) to garner leadership, champion, and provider support to adopt, implement, and programmatically sustain ACP-GV and (2) to recruit, deploy, and spread ACP-GV to a diverse representation of veterans and those they trust engaged in groups that may be offered throughout VA healthcare (e.g., whole health wellness groups, caregiver support groups, psychosocial recovery groups, etc.). Finally, the stages of program implementation are presented as four phases: Exploring, Adopting, Implementing and Sustaining. In order to be an active ACP-GV site, site leads are expected to complete a set of activities within each phase, progressively moving from one phase to the next, ultimately reaching sustainment as noted in Table 1. A new clinical tool recently fielded provides descriptions and prompts for the site leads to successfully move their developing program from exploring to sustainment. These two trainings ensure that facilitators and site leads have the ACP-GV background necessary to be successful in implementation of the program locally. The next section details the development of evaluation metrics to assess the program's success nationally.

**Table 1:** National ACP-GV program implementation components by phases and core elements.

Phase	Core elements			
Exploring	Identify local ACP-GV point of contact	Obtain ACP-GV knowledge	Assess your site	Engage stakeholders and leadership
Adopting	Complete ACP-GV national training	Formalize commitment & build infrastructure	Develop outreach & dissemination plan	Promote the program
Implementing	Conduct ACP-GV groups	Track and assess implementation	Engage stakeholders & leadership	Prepare to sustain program
Sustaining	Integrate program into health system	Identify permanent staff	Crosstrain & develop coverage plan	Hold groups in multiple areas with multiple facilitators

### Advance care planning via group visits adoption of the RE-AIM framework

As noted earlier, the National ACP-GV Program adopted RE-AIM early, prior to the request from ORH. The adoption occurred as part of a grant award for additional intramural funding from the VA's Quality Enhancement Research Initiative (QUERI) (11). Given the focus on evidence-based practices, evaluation, and implementation strategies that is required for this type of funding, reviewers specifically requested the use of conceptual and analytic frameworks to support the selection of measured constructs. RE-AIM was chosen as the analytic framework.

With the addition of this funding devoted to evaluation, ACP-GV gathered a diverse team of program developers, field-based implementation staff, evaluators, data analysts, and veterans. The team worked collaboratively to propose and develop a variety of metrics for the QUERI-funded national program evaluation. This inclusive method of gathering ideas on meaningful data from various stakeholders, particularly for estimating time devoted to implementation, was unique for a planned budget impact analysis (12). It was during this time that ORH encouraged the use of RE-AIM metrics in annual reports, provided training and technical assistance on the use of RE-AIM, and integrated an annual review and feedback process for requesting refinements to the metrics. With ample funding from both ORH and QUERI and a focus on evaluation, the National ACP-GV Program easily accommodated both funders' request to use RE-AIM. As such, our aims for this article are to describe: (1) how RE-AIM was used to evaluate ACP-GV and (2) lessons learned from ORH's annual reporting feedback to ACP-GV on the use of the framework to describe evaluation impacts.

## Materials and methods

This section describes the development and use of RE-AIM in the National ACP-GV Program and then presents the results of using these metrics for ORH's annual reporting purposes. Following this, in the discussion section, we describe lessons learned and implications for considering how this approach to a national evaluation of a program might then be translated to making local improvements in clinical settings or even be leveraged to advocate for the use of the ACP-GV intervention in other settings.

### Data sources

Patient- and provider-level administrative health care data was obtained from the VA Corporate Data Warehouse and national program management databases for federal fiscal years (FY) spanning October 1, 2018 to September 30, 2023. These six years correspond to the first full year of ORH funding awarded to the National ACP-GV Program and the overlap of four years of QUERI funding.

### Setting and sample

The setting for this evaluation is VA healthcare. More specifically, a VA site, defined as a VA hospital or healthcare system that includes primary, acute, and residential care settings. Within VA healthcare, community based outpatient clinics are networked to these major VA hospitals which comprise a broader geographic area in their respective healthcare system. For our evaluation, we use the total of 172 VA facilities as the total number of potential sites (13).

In terms of the sample, sites were further organized as one of three types. The types relate to funding status. As background, as part of ORH funding to the National ACP-GV Program, sites who wished to adopt ACP-GV and agreed to focus efforts on serving rural veterans and/or rural areas could apply for three years of ORH seed funding to support clinical and administrative staff delivering groups. These sites main function was to provide ACP-GV in their VA's respective network of community based outpatient clinic settings, with particular attention to rural areas and veterans. The goals were to increase access to, and the efficiency of, advance care planning with rural veterans by using the group modality. Against this background, the QUERI funding was a compliment to the ORH funding; the QUERI funding expanded the evaluation to include all sites who wanted to adopt ACP-GV but did apply or receive ORH seed funding. The resulting three types of sites based on funding status include (1) ORH funded sites, (2) sites who complete the three years of ORH funding, referred to herein as “post-funded sites”, and (3) sites without any ORH funding referred to herein as “unfunded sites”. The focus on disaggregating the impact on rural veterans and service delivery in rural settings was emphasized as part of the mandate for ORH funding when RE-AIM was first introduced as the chosen analytic framework. In this evaluation, all of the rural specific metrics were added over time and based on ORH annual reporting feedback.

### Metrics

**Figure 1:**
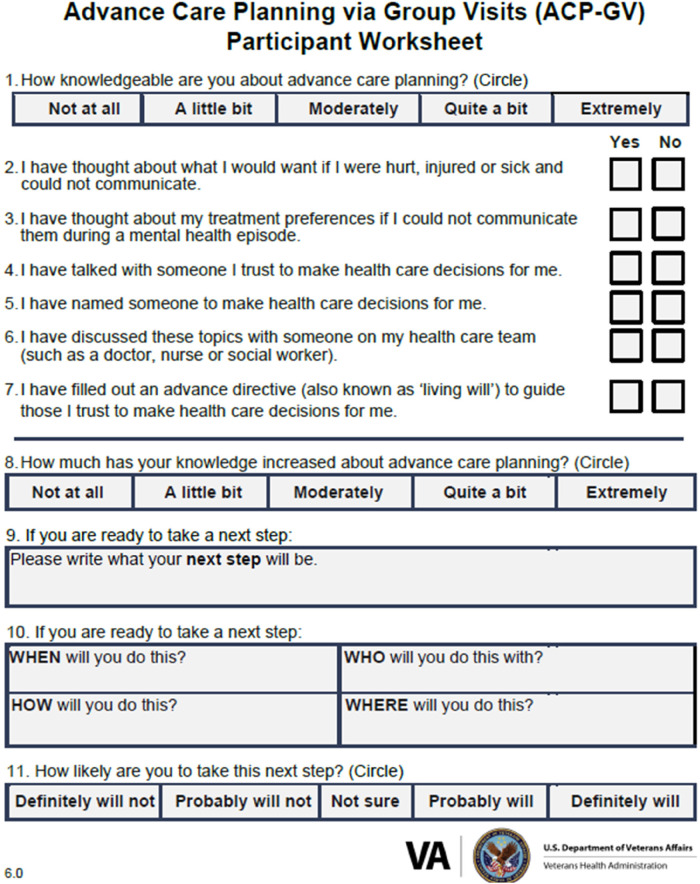
Advance care planning via group visits worksheet.

Measures for this program evaluation included the following RE-AIM metrics. *Reach* is defined as the cumulative number of unique veterans (e.g., first time users enrolled in VA healthcare), rural veterans, rurality percentage, and demographics of veterans served by ACP-GV from program inception and in the past FY obtained from the advance care planning notes report curated by the Veterans Support Service Center (VSSC). *Effectiveness* is defined as the percentage of veterans with increased knowledge from items obtained from the ACP-GV Participant Worksheet administered during ACP-GV in the past FY (see Figure 1). These knowledge items correspond to health factors derived from participation in ACP-GV which are entered by clinical staff as part of documentation of visits in VA electronic health records. At the beginning of group, participants are prompted by the facilitator to answer questions 1–7 on the worksheet. This document is also used at the end of the group to assess knowledge gained and to support each individual participant in goal setting.

*Adoption* is defined at the site- and provider-level. First, adoption is measured by the cumulative number of VA facilities adopting ACP-GV, disaggregated by sites who were awarded three years of ORH seed funding to isolate these sites by funding status. Post-funded sites received three years of ORH seed funding and continued to provide ACP-GV after the conclusion of seed funding.

Second, for providers, the cumulative number of VA staff trained annually is calculated from reports curated by the National ACP-GV Program database. The National ACP-GV Program provides a live, instructor-led training monthly (i.e., Facilitator Training or Site Lead Training). In the first quarter of FY 2022, attendance data was monitored and collected during monthly trainings by program staff. Attendees must have been present each hour of the three-hour training to be considered present by the National ACP-GV Program. In the last three quarters of FY 2022, attendance data was collected by self-report using an online registration data collection system and provided to the National ACP-GV Program through a report provided by the Institute for Learning, Education and Development. These data were merged into one analytic database.

With regard to implementation adherence to the ACP-GV model, group facilitators administer the ACP-GV Participant Worksheet to participants at the beginning and collect it at the end of the group, as previously mentioned. Use of the worksheet is a hallmark of distinguishing ACP-GV from other non-ACP-GV approaches. The questions asked on the worksheet provide a framework for the ACP-GV discussion and responses are documented within the electronic health record of participants. *Implementation* is measured by the number and percent of veterans completing this worksheet administered during ACP-GV in the past FY, which indicates provider adherence to the program model and veteran engagement in knowledge assessments regarding advance care planning during the group.

*Maintenance* is defined as the ability to continue to deliver ACP-GV following the completion of the three-year funded period for ORH funded sites and one year following implementation for sites not funded by ORH.

### Data analysis

Frequencies and percentages were used to summarize key demographic characteristics and metrics from veterans and non-veterans (e.g., caregivers) who participated in ACP-GV, site- and provider-level information, and national program metrics using the RE-AIM constructs.

## Results

### Evaluation findings

RE-AIM metrics are described in Table 2 for cumulative and the past FY 2023. Reach is simply defined as the cumulative and annual number of unique veterans and rural veterans that attended ACP-GV. To date, this project has reached 54,168 veterans, including 19,032 unique rural veterans. During FY 2023, 7,451 veterans participated in ACP-GV, with 2,788 being from rural or highly rural areas. Sites funded by ORH in FY 2023 served a total of 2,190 participants through ACP-GV, 744 of which were from rural or highly rural areas. Finally, the cumulative percentage for rurality was calculated for all participants. In FY 2023, ACP-GV's participation rate for serving rural veterans was 38.2%.

**Table 2:** National ACP-GV program *reach* metrics from federal fiscal years spanning October 1, 2018 to September 30, 2023.

Fiscal year (FY)^a^	ACP unique patients	ACP-GV unique veterans each FY	ACP-GV unique rural veterans each FY	ACP-GV percent rural veterans each FY
2018	637,865	11,490	4,277	37.2%
2019	628,425	13,005	4,888	37.6%
2020	503,815	7,038	2,268	32.2%
2021	534,411	8,697	2,550	29.3%
2022	538,786	6,981^b^	2,261	32.4%
2023	618,288	6,939^c^	2,649	38.2%

^a^ Veterans can have visits in multiple fiscal years.

^b^ FY 2022 non-veteran, i.e., caregivers, (*N* = 207) were removed from the ACP-GV unique veteran ACP-GV total.

^c^ FY 2023 non-Veteran, i.e., caregivers, (*N* = 308) were removed from the ACP-GV unique Veteran total.

Effectiveness is defined as the percentage increase in veteran knowledge and confidence after attending ACP-GV. Of the 7,451 participants who completed an ACP-GV Participant Worksheet during the group in FY 2023, 58% reported an increase in knowledge (a little bit\moderately\quite a bit\extremely) about advance care planning and 14% reported a substantial increase (quite a bit\extremely). At the end of group, as part of their clinical documentation, the facilitator records the veterans' responses from the worksheet within the electronic medical record.

For adoption, the metrics include the cumulative total number of adopting sites as well as the cumulative number of providers trained in ACP-GV. For site adoption (see Table 3) at the end of FY 2023, ACP-GV was adopted by 66 sites. When disaggregated, this totals 12 ORH-funded sites, 27 post-funded sites, and 27 unfunded sites. Fifteen sites completed the three-year funding cycle at the end of FY 2022. For staff adoption, since program inception in FY 2017, 1,556 staff have been trained to facilitate ACP-GV groups.

**Table 3:** National ACP-GV program local site a*doption* numbers (*N* = 66).

Active sites				
Fiscal year	Office of rural health funded sites		Unfunded sites	Total
	Seed funded	Post funded		
2017^a^	18	—	1	19
2018	26	—	3	29
2019	23	—	17	40
2020	38^b^	—	17	55
2021	26	15	16	57
2022	25^c^	17	21	63
2023	12	27	27	66

—Not applicable until after 3-years of funding.

^a^ Partial year funding due to out-of-cycle application for initial launch.

^b^ Includes partial year FY 2017 sites whose 3-year funding cycle concluded mid-FY 2019.

^c^ Three sites held groups but withdrew from funding.

During FY 2023, implementation adherence, which indicates provider adherence to the program model and veteran engagement in knowledge assessments regarding advance care planning during the group, was noted in 91.7% (*n* = 6,589) of program completers (See Table 4). Finally, for maintenance, of the 66 active sites, 27 post-funded sites have continued to offer ACP-GV in successive years after the conclusion of their receipt of three years of ORH seed funding. Clearly, sites without seed funding are sustaining ACP-GV.

**Table 4:** *Implementation* metrics for adherence to the ACP-GV model during FY 2023: responses to the ACP-GV worksheet documented in the electronic health record (*n* = 7,188).

First ACP-GV visit FY 2023			
Worksheet recorded		Yes (%)	No (%)
Number of participants		6,589 (91.7%)	599 (8.3%)

Missing data: 599 participants did not have worksheet responses documented.

## Discussion

This manuscript described the evaluation of the National ACP-GV Program using RE-AIM metrics. VA administrative and national program databases provided data to measure the cumulative and past FY metrics developed to assess Reach, Effectiveness, Adoption, Implementation, and Maintenance of ACP-GV between the years 2018–2023. In this timeframe, ACP-GV *reached* 54,168 unique veterans, including 19,032 unique rural veterans, and 1,556 VA staff *adopted* the practice as evidenced by completion of training. During FY 2023, *implementation* adherence to the ACP-GV model was noted in 91.7% of program completers, with 55% of these completers reporting knowledge about ACP increase and 14% reporting a substantial knowledge increase (*effectiveness*). Finally, 66 sites *adopted* ACP-GV, but more importantly 27 previously funded sites *maintained* ACP-GV after ORH seed funding ended. To date, ACP-GV is active in over a third (66/172) of VA facilities across the nation within six years. In the following section, we will describe lessons learned from ORH's annual reporting feedback on ACP-GV's use of the RE-AIM framework to describe their evaluation impacts.

### Implications for practice, policy, and evaluation

Challenges and opportunities in using RE-AIM for the national program and the funder are offered for consideration by other large healthcare systems, funding organizations, research and development departments, and evaluation focused organizations. There were a number of considerations when adopting RE-AIM as the analytic strategy in health care, research, evaluation, and other public health settings (14). Strengths for ACP-GV as the national program and ORH as the funder in using RE-AIM include providing common metrics of interest for the national project and the funder, a more focused annual report, and enhancing comparability of similar data across projects in the larger portfolio for the funder. In many ways, the main challenges at the funder-level include the need for a higher level of evaluation experience and expertise needed on the projects or increased training and technical assistance to project staff, the differing definitions of each RE-AIM construct, and that existing data may not be available, systematically collected, or easy to access, interpret, or fit the RE-AIM constructs or fit easily for varying types of Enterprise-Wide Initiatives in the ORH portfolio (e.g., workforce development projects).

For the National ACP-GV Program, RE-AIM began with a requirement from QUERI funders for the use of these metrics. Unfortunately, the evaluators working with the program developers and implementers did not have the long program history and vast clinical wisdom to realize that some proposed metrics would not be achievable. For example, evaluators proposed that reach be measured by the proportion of veterans impacted by ACP discussions divided by all veterans seeking care in VHA facilities in a FY. This metric is problematic as the national mandate for ACP notes that every VA must *offer* ACP to veterans and document their response. The actual delivery of ACP-GV is optional. Therefore, not all veterans *receive* ACP.

From this experience, the evaluation team shifted from developing metrics *for* the National ACP-GV Program to a more collaborative creation of the RE-AIM metrics *with* the program developers and implementers. Each year since, in preparing the annual report, the full team meets to review existing data available to the National ACP-GV Program and discuss how to evaluate the data quality of each prospective metric. Once the analysts review the data sources and calculations, a smaller workgroup review the annual reporting requirements for QUERI and ORH funders noting similarities in the definitions and requirements to report metrics for reach and adoption (i.e., number of veterans reached, number of providers trained, and the number of VA facilities adopting). Annual totals were quickly developed for reach and adoption that soon led to the calculation of cumulative numbers for all metrics since program inception.

At the same time, technical assistance on the use of RE-AIM and supportive feedback from reviewing annual reports helped overcome the initial challenges with the metrics proposed in the QUERI grant. The National ACP-GV Program now has two evaluation reporting leads that prepare preliminary drafts of annual reports that are then reviewed by the analysts and evaluators for data quality and consistency of reporting. Each year, new metrics have been added from feedback received from the funders' review of the previous year's report. ORH has increasingly advocated for the inclusion of new or more specific metrics (e.g., rural specific numbers and disaggregation), some of which cause changes in the data sources or revised calculations of particular metrics. Each change noted in the annual report has a number of caveats as to the methodological consequences and, for some, the unresolved barriers are noted. Overall, segregating each funder's contribution to the program by disaggregating the data adds analytic burden, can make interpretation challenging, and may not be readily available without ongoing evaluation support. In summary, lessons learned developing RE-AIM metrics collaboratively with program developers, implementers, and evaluators allows for a balance of clinical and scientific input in decision-making, while the ORH annual reporting feedback provided specificity and emphasis for including cumulative, annual, and rural specific metrics.

Finally, our experience using RE-AIM to frame a national evaluation of a program can offer additional advantages for development and use in other settings beyond VA. Experience with developing simple reach and adoption metrics can provide clear indications of how many individuals, staff, and sites are involved in a practice before, during, or after a planned change effort. This temporal aspect to data collection can provide additional time points for comparison, opportunities to estimate the gaps in care, and may be useful with a variety of clinical outcomes. Effectiveness and implementation can be challenging concepts for some new to metric development or program implementation, so definitions, case examples, and creative approaches to analyzing, visualizing, and summarizing data can be extremely helpful. Lastly, maintenance metrics typically require a time element as well, once it is determined what constitutes the end of implementation. In all cases, a pilot test of the metrics is highly recommended to adjust course when the proposed metrics do not add value or provide clear and meaningful program impacts.

When consulting with clinical managers and health care executives from non-VA healthcare settings, we share our experiences and guidelines for not only program implementation but also provider documentation templates and clinic set up guides. It is common to provide technical assistance during the adoption stage and/or to strategize how to capture patient encounters in differing electronic health record systems earlier during exploration.

The use of RE-AIM metrics in summarizing program-level outcomes may assist clinical managers to establish a baseline and future goals when making local improvements to practices in their clinical settings. Healthcare executives could also leverage VA data from our evaluation to advocate for the adoption of our ACP-GV intervention in their settings. Regardless, both ACP-GV and its use of RE-AIM show the program's impact on veterans. These lessons learned are meant to offer general information and examples that may be useful to other healthcare systems that could benefit from offering a patient-centered group approach to advance care planning.

### Limitations

This evaluation is not without limitations. Some national metrics proposed in previous years have not been continued. For adoption, the number of VA staff trained has not been collected in relation to the employees' site or rurality. Efforts to collect this data will begin in FY 2024; however, some initial challenges to adding demographics to the online course delivery system will limit the start of this data collection, and archival data cannot provide what it does not have. Another limitation related to alternative implementation metrics considered but not used are noted. As part of the National ACP-GV Program, all sites are oriented to the ACP-GV Fidelity Instrument. The Fidelity Instrument serves as the reference tool supporting fidelity to the intended model of the ACP-GV session. The Fidelity Instrument aligns to the program's training materials, including a video of the ACP-GV intervention that uses actors to show the intended design of group and relays the structure and flow of group along with prompts for key skills and content. Sites are instructed to use the Fidelity Instrument to train new group facilitators and to identify learning opportunities for existing group facilitators. Site-level data from completed Fidelity Instruments is not collected nationally due to several barriers, including lack of automation and budgetary restrictions that limit the provision of ongoing technical assistance. Finally, typically, cost evaluations are included as a part of implementation metrics; however, for ACP-GV, results of the budget impact analysis are forthcoming and may be considered for future annual report metrics.

## Conclusions

There are challenges and opportunities to using RE-AIM for funders and programs. Nevertheless, ACP-GV's use of RE-AIM is a key step towards improving rural veteran health outcomes and describing real world program impacts of advance care planning with veterans.

## Data Availability

All raw data are the property of the United States Government; data availability will be subject to review by the Department of Veterans Affairs and must be in compliance with all applicable federal policies and laws.
